# Changes in microbial population and chemical composition of corn stover during field exposure and effects on silage fermentation and *in vitro* digestibility

**DOI:** 10.5713/ajas.18.0514

**Published:** 2018-11-27

**Authors:** Lin Sun, Zhijun Wang, Ge Gentu, Yushan Jia, Meiling Hou, Yimin Cai

**Affiliations:** 1 Key Laboratory of Grassland Resources, Ministry of Education, P.R. of China, College of Grassland, Resources and Environment, Inner Mongolia Agricultural University, Hohhot 010019, China; 2 Inner Mongolia Academy of Agricultural Sciences & Animal Husbandry, Hohhot, Inner Mongolia, 010030, China; 3 Inner Mongolia Institute of Grassland Surveying and Planning, Hohhot, Inner Mongolia 010051, China; 4 College of Agriculture, Inner Mongolia University of Nationalities, Tongliao 028000, China; 5 Japan International Research Center for Agricultural Science (JIRCAS), Tsukuba, Ibaraki 305-8686, Japan

**Keywords:** Chemical Composition, Corn Stover, Digestibility, Field Exposure, Silage Fermentation

## Abstract

**Objective:**

To effectively use corn stover resources as animal feed, the changes in microbial population and chemical composition of corn stover during field exposure, and their silage fermentation and *in vitro* digestibility were studied.

**Methods:**

Corn cultivars (Jintian, Jinnuo, and Xianyu) stovers from 4 random sections of the field were harvested at the preliminary dough stage of maturity on September 2, 2015. The corn stover exposed in the field for 0, 7, 15, 30, 60, 90, and 180 d, and their silages at 60 d of ensiling were used for the analysis of microbial population, chemical composition, fermentation quality, and *in vitro* digestibility. Data were analyzed with a completely randomized 3×6 [corn stover cultivar (C)×exposure d (D)] factorial treatment design. Analysis of variance was performed using SAS ver. 9.0 software (SAS Institute Inc., Cary, NC, USA).

**Results:**

Aerobic bacteria were dominant population in fresh corn stover. After ensiling, the lactic acid bacteria (LAB) became the dominant bacteria, while other microbes decreased or dropped below the detection level. The crude protein (CP) and water-soluble carbohydrate (WSC) for fresh stover were 6.74% to 9.51% and 11.75% to 13.21% on a dry matter basis, respectively. After exposure, the CP and WSC contents decreased greatly. Fresh stover had a relatively low dry matter while high WSC content and LAB counts, producing silage of good quality, but the dry stover did not. Silage fermentation inhibited nutrient loss and improved the fermentation quality and *in vitro* digestibility.

**Conclusion:**

The results confirm that fresh corn stover has good ensiling characteristics and that it can produce silage of good quality.

## INTRODUCTION

Corn (*Zea mays* L.) is an important crop for food production in China and worldwide. Corn stover includes the leaves, stalks, husks, and cobs, and is often reported as a ratio of dry matter (DM) of corn grain to residue of 1:1. Corn stover is the main crop residue in most of China, with an annual yield of approximately 640 million t/yr [1]. Using corn stover as animal feed has been proven economically viable, not only as a method of disposing of corn stover residue, but also as an alternative livestock feed in regions where corn is the main crop [1]. Corn stover is sometimes incinerated in the field [2], raising concerns about air pollution, fine particle generation, and global warming. Moreover, corn stover is considered an important local resource that can be used by farmers to prepare silage as an animal feed and as a rural fuel [3]. Not only fresh corn stover but also its silage is used as an animal feed; large amounts of dried stover are fed to dairy or beef cattle in some localities, China. However, the dry corn stover that has been left exposed in the field for a long time is very fibrous, low in nutrients and difficult to digest for animals [1]. Feeding livestock low-quality roughage can result in low production. The major constraint for livestock production in some cold climates, such as Inner Mongolia, is a shortage of good-quality feed, where corn stover or hay is the major source of roughage for livestock in winter. Fresh corn stover has good ensiling characteristics due to its relatively high DM content at harvest, low buffering capacity, and adequate water-soluble carbohydrate (WSC) content [4,5]. Corn stover silage has become the major component of forage for dairy cows under most dietary regimes in China [2,6], and has greater potential to improve ruminant production than the more conventional preserved forages used in corn production areas [7].

Silage preparation and storage are the most effective prepa ration techniques for fresh stover [8,9]. Corn stover silage can be beneficial to dairy cattle, beef cattle, or sheep producers because it can provide a locally available feed source at a low cost for animal production [1], and silage is a commonly preserved feed in many countries, including China. The preservation of forage crops as high-quality silage depends on the production of enough acids to inhibit the activity of undesirable epiphytic microorganisms under anaerobic conditions, especially lactic acid bacteria (LAB), which are naturally present on forage crops [10,11].

However, most studies in silage research have analyzed silage fermentation of fresh corn stover, and limited information is available on the effects of field drying corn stover on feed composition, silage fermentation, and digestibility. In this study, the changes in the microbial population and chemical composition of corn stover during field exposure for up to 180 d and their silage fermentation characteristics were studied. In order to evaluate the nutritional value of the corn stover and silages, we also studied the *in vitro* digestibility of the stover and silage in a corn production area of Inner Mongolia, China.

## MATERIALS AND METHODS

### Animal care

Animal experiments were approved by the Committee of Animal Experimentation and were performed under the institutional guidelines for animal experiments of the College of Grassland, Resources and Environment, Inner Mongolia Agricultural University, China. The experiments were performed according to recommendations proposed by the European Commission (1997) to minimize the suffering of animals.

### Corn stover and silage preparation

Local corn (*Zea mays* L.) cultivars (Jintian, Jinnuo, and Xianyu) were obtained from an experimental field at the Inner Mongolia Agricultural University (Huhhot, China). Corn stovers from 4 random sections of the field were harvested at the preliminary dough stage of maturity on September 2, 2015. Silage was prepared from stover exposed in the field for 0, 7, 15, 30, 60, 90, and 180 d. The ensiling material in triplicate for each cultivar was cut into 20-mm segments with a chopping machine (TS420; Qufu Machinery Equipment Co., Ltd., Qufu, China), and adequate water added to get a moisture content of approximately 60%. Silage was fermented in laboratory-scale polyethylene silos (1-L volume, 10-cm diameter, 20-cm length; Shenzhen Guanruilong Chemical Co., Ltd., Shenzhen, China), at approximately 760 g (fresh weight). The silos were sealed with a screw top and plastic tape and stored at ambient temperature (20°C to 25°C). The silos were opened after 60 d of ensiling, and the microbial composition, chemical composition, fermentation quality, and *in vitro* digestibility were analyzed.

### Microbiological and chemical analysis

Corn stover and silage samples (10 g) were blended with 90 mL of sterilized water, and serially diluted in sterilized saline solution (8.50 g/L NaCl) from 10^−1^ to 10^−5^ [12]. The number of LAB was determined using the plate-counting method on MRS agar (Difco Laboratories, Inc., Detroit, MI, USA) incubated at 30°C for 48 h in an anaerobic box (MGC C-31; Mitsubishi Gas Chemical Co., Inc., Tokyo, Japan). Yeasts and molds were counted on potato dextrose agar (Nissui Ltd., Tokyo, Japan) after incubation at 30°C for 96 h, and aerobic bacteria were counted on nutrient agar medium (Qingdao Hope Bio-technology Co., Ltd., Qingdao, China). All microbial data were transformed to log10 colony-forming units (cfu) based on fresh matter (FM).

The DM contents of corn stover and silage was determined by weighing samples that had been oven-dried at 65°C for 48 h, and the data were corrected for residual moisture at 105°C [13]. The crude ash (CA) content was determined after placing samples in a muffle oven for 3 h at 550°C according to the method of No.942.05 of the AOAC [14]. The organic matter (OM) was calculated as weight loss upon ashing. Total nitrogen was measured using a Kjeldahl apparatus (KDY-9830, Shanghai, China) using the procedure of the AOAC [14]. Crude protein (CP) was calculated as follows: total nitrogen×6.25. Neutral detergent fiber (NDF) and acid detergent fiber (ADF) contents were determined using the ANKOM A200i fiber analyzer (ANKOM Technology, Macedon, NY, USA) [15]. The ether extract (EE) content was determined according to procedure No.963.15 of the AOAC [14]. The WSC concentration of fresh materials and silages were determined using the method of Kim and Adesogan [16].

For the silage fermentation analysis, 10 g of sample was homogenized with 90 mL of deionized water for 5 min [17]. The extract was filtered through two layers of cheesecloth and a filter paper (8 cm; Aoke Co., Ltd., Taizhou, China), and the pH was measured using a glass electrode pH meter (STARTER 100/B; OHAUS, Shanghai, China). The ammonia nitrogen (N) content was analyzed using a steam distillation method of the filtrates, and lactic, acetic, propionic, and butyric acids were analyzed by high-performance liquid chromatography, as described by Cai [17].

### *In vitro* incubation and degradability measurements

*In vitro* fermentation was performed in serum bottles following the method described by Contreras-Govea et al [18] and Kowalski et al [19]. Rumen fluid was obtained before morning feeding from four Inner Mongolia semi-fine-wool sheep (wethers) through stomach tube. Animals were fed 40 g of alfalfa hay, 400 g of guinea-grass hay, and 240 g of concentrate containing 10% wheat bran, 25% soybean meal, 65% corn grain, and supplemental vitamins and minerals on a DM basis.

Approximately 1 g ground sample (through 1 mm sieve) was placed in 130-mL serum bottles. Rumen fluid was filtered through four layers of gauze and mixed 1:2 (v/v) to buffer; 60 mL of the mixture was transferred into each serum bottle. The buffer was prepared by the method described by Menke [20] and consisted of (added in order) 400 mL H_2_O, 0.1 mL solution A (13.2 g CaCl_2_ ·2H_2_O, 10.0 g MnCl_2_·4H_2_O, 1.0 g CoCl_2_·6H_2_O, 8.0 g FeCl_3_·6H_2_O and made up to 100 mL with H_2_O), 200 mL solution B (39 g NaHCO_3_/L, H_2_O), 200 mL solution C (5.7 g Na_2_HPO_4_, 6.2 g KH_2_PO_4_, 0.6 g MgSO_4_·7H_2_O and made up to 1,000 mL with H_2_O), 1 mL resazurine (0.1%, w/v) and 40 mL reduction solution (95 mL H_2_O, 4 mL l N NaOH and 625 mg Na_2_S·9H_2_O). Each serum bottle was kept under CO_2_ in a water bath at 39°C, after being capped with a butyl rubber stopper and sealed with an aluminum crimp. In the experiment, the gas production (GP) was released and measured during *in vitro* incubation at every 4 hours’ point intervals. The cumulative 48 h GP data was the total amount of gas produced every 4 hours. Immediately after 48 h incubation, the undigested solids were precipitated by centrifugation at 1,000×*g* for 10 min at room temperature and dried in an aerated oven at 65°C for 48 h, and were then assayed for DM and CA. The *in vitro* degradability of OM (OM-D) was calculated as change in its respective weight. All corn stover and their silages were performed in two separate *in vitro* experimental runs, and each run consisted of 132 bottles: three cultivars×seven exposure d×three replicates×2 (corn stover+its silage), plus six blanks. The blanks were the same bottle with no materials. The cumulative 48 h GP was corrected by subtracting GP from blank bottles.

Ruminal pH was measured immediately after collection using a digital pH meter (STARTER 100/B; OHAUS, China), and a 20-mL sample was preserved with 0.5 mL of 9 M sulfuric acid and stored at −20°C for subsequent analysis of total volatile fatty acids (VFAs). The total VFA concentration was measured using an automated gas chromatograph [21].

### Statistical analysis

Data on the microorganism population, chemical composition, and fermentation quality after 60 d of ensiling and *in vitro* digestibility were analyzed with a completely randomized 3×6 (corn stover cultivar [C]×exposure d [D]) factorial treatment design. Analysis of variance was performed using SAS ver. 9.0 software (SAS Institute Inc., Cary, NC, USA), and the statistical model is as follows:

$${\text{Y}}_{\text{ijk}}=\mu +\alpha_{\text{i}}+\beta_{\text{j}}+\alpha \beta_{\text{ij}}+{ɛ}_{\text{ijk}}$$

Where Y _ijk_ is the observation, μ is the overall mean, α_i_ is the effect of corn stover cultivar (i = Jintian, Jinnuo, and Xianyu), β_j_ is the effect of exposure d (j = 0, 7, 15, 30, 60, 90, and 180), αβ_ij_ is the effect of corn stover cultivar×exposure d, and ɛ_ijk_ is the error. The mean values were compared using Duncan’s test [22].

## RESULTS

Table 1 presents the chemical composition of corn stover during the field exposure. At 0 d of exposure, the three cultivars had similar DM contents (30.21% to 30.67%), which increased by 4% to 25% after 7 to 180 d of exposure. The CP contents of the three cultivars were 6.74% to 9.51% of DM. After 180 d of exposure, the CP content decreased to 3.28% to 4.41% of DM. The NDF and ADF contents in the three cultivars were 43% to 61% and 27% to 38% of DM, respectively. The exposure d means in the three cultivars were similar at 7 and 30 d, and differed significantly (p<0.05) for the other exposure d. With the increase of exposure time, the WSC content decreased markedly, and the mean WSC in the three cultivars decreased sequentially with increasing exposure time (p<0.05). C, D, and C×D influenced (p<0.0001) the DM, OM, CP, NDF, ADF, and WSC contents. C and D influenced (p<0.0001, p = 0.0006) the EE content, but C×D did not (p = 0.999).

Table 2 presents the microbial population of corn stover over the course of the field exposure. Fresh (0 d of exposure) Jinnuo, Jintian, and Xianyu corn stover contained 10^4^ to 10^8^ cfu/g of FM of LAB, aerobic bacteria, yeasts, and molds. From 7 to 180 d of exposure, these microorganism counts of the three cultivars were 10^4^ to 10^7^ cfu/g of FM. The mean aerobic bacteria and molds counts of Jinnuo and Jintian at 180 d of exposure were significantly (p<0.05) lower than other exposure days. C, D, and C×D influenced (p<0.0001) the counts of all microorganisms.

Table 3 presents the microbial population of corn stover silage after 60 d of ensiling. The LAB counts in the silages of the three cultivars were 10^4^ to 10^7^ cfu/g FM. The mean LAB counts of the silages of the three cultivars from the first few d of exposure (0, 7, and 15 d) were significantly (p<0.05) higher than those of silages after 30, 60, 90, and 180 d of exposure. During exposure, the aerobic bacteria and yeasts remained around <10^3^ to 10^7^ cfu/g of FM, and molds in all silages were below the detection level (<10^3^ cfu/g of FM). C, D, and C×D influenced (p<0.0001) the counts of LAB, aerobic bacteria, and yeasts in corn stover silage.

Table 4 presents the chemical composition of corn stover silage after 60 d of ensiling. The OM contents of the three cultivar silages after 0 to 180 d of exposure were similar, ranging from 93% to 95% of DM. With the increase of exposure time, the CP contents of all corn stover silages significantly (p<0.05) decreased. The highest (p<0.05) and lowest (p<0.05) CP contents in the three cultivars were obtained at the initial (0 d) and final (180 d) exposure times. During exposure, the mean NDF and ADF contents in the silages of the three cultivars were 46% to 51% and 26% to 33% of DM, respectively; the EE content decreased gradually (p<0.05). With the increase of exposure time, the WSC content of the silages of the three cultivars, as well as the mean decreased (p<0.05). The C, D, and C×D influenced (p<0.0001 or p = 0.0017) the OM, CP, EE, NDF, ADF, and WSC contents. C and D influenced (p<0.0001, p = 0.0017, respectively) EE, but C×D did not (p = 1.0000).

Table 5 presents the fermentation quality of the corn stover silages after 60 d of ensiling. The silages of the three cultivars from corn stover exposed for 0 and 7 d were well preserved, with lower (p<0.05) pH and ammonia-N contents and higher (p<0.05) lactic acid content than those of the silages from stover exposed for 15 to 180 d. The silages prepared from corn stover exposed for 180 d yielded the worst fermentation quality (p< 0.05). Jintian stover had a significantly higher mean of lactic acid content (p<0.05) and lower mean ammonia-N content (p<0.05) than the silage from the other two cultivars. The C, D, and C×D influenced (p<0.0001) the pH and contents of lactic acid, acetic acid, propionic acid, and ammonia-N. The C did not influence (p = 0.2579) the butyric acid content of corn stover silage, but the D (p = 0.025) and C×D did (p = 0.0038).

Table 6 presents the ruminal pH, GP, total VFA content, and OM-D of corn stover and silage. The ruminal pH of the three cultivars at different exposure times were similar (6.12 to 6.30). With the increase of exposure time, the GP, total VFA content, and OM-D of the three cultivars decreased. The highest (p<0.05) and lowest (p<0.05) GP, total VFA content, and OM-D in the three cultivars were observed in stover on the initial (0 d) and final (180 d) d of exposure, respectively. The C and D influenced (p<0.0001) GP, total VFA content, and OM-D. C×D influenced (p = 0.0028) GP but did not influence (p = 0.0816) VFA content or OM-D (p = 0.1884). The A comparison of the three cultivars showed that the GP and total VFA content were highest (p<0.05) in Jintian corn stover. The OM-D of Jinnuo corn stover was higher (p<0.05) than the other cultivars.

The ruminal pH of the stover silages of the three cultivars at different exposure times ranged from 6.61 to 6.86. With the increase of exposure time, the GP, total VFA, and OM-D of the three cultivar silages decreased. Compared to the data from stover collected on day 0, the mean GP, total VFA content, and OM-D of corn stover silage after 180 d of exposure decreased significantly (p<0.05) by more than 5%, 11%, and 6%, respectively. The C and D influenced (p<0.0001) GP, total VFA content, and OM-D. C×D influenced (p = 0.0004) OM-D but did not influence (p = 0.7809) GP or total VFA (p = 0.0647) content. From the cultivar means, the OM-D of Jintian corn stover silage was significantly (p<0.05) higher than those of Xianyu and Jinnuo corn stover silage.

## DISCUSSION

Corn stover is an abundant byproduct of corn grain harvesting. Due to economic and environmental concerns, there is an increasing demand for the efficient use of crop residues, including corn stover. Farmers use fresh or dry corn stover as forage to feed ruminants. However, dry corn stover is highly fibrous and difficult to digest when stored for long periods on fields. Therefore, it is important to identify techniques to effectively use corn stover. After drying on fields, the moisture and CP contents of corn stover decrease and the NDF content increases [23]. In this study, the DM of the three cultivars increased by more than 4% to 25% after 7 to 180 d of exposure. Generally, CP includes true protein and non-protein nitrogen such as urea nitrogen and ammonia nitrogen. The CP content is influenced by exposure condition and activity of the protein-degrading microorganisms. In the present study, after 180 d of exposure, the CP contents of corn stover were decreased by 34% to 66% compared to fresh stover or their silages. The reason is that the rain, exposure, and microbial populations may result in the CP loss, and that the good quality of silage could effectively preserve these chemical compositions of stover during ensiling. Aerobic bacteria, yeasts, and molds were distributed in fresh corn stover at concentrations of 10^4^ to 10^8^ cfu FM (Table 1). The mean aerobic bacteria and molds counts of Jinnuo and Jintian at 180 d of exposure were significantly (p<0.05) lower than other exposure days. The reason for this difference is probably due rain before sampling, and epiphytic microorganisms may have consumed some of the chemical components of the corn stover, such as CP and WSCs.

Meanwhile, the NDF and ADF contents of dry corn stover did not show specific trends during field exposure, which differed from the results of previous studies, where the crude fiber content was greater in dry corn stover than fresh stover [23]. The reason for this difference is unclear; however, exposure to rain and natural conditions may have caused a loss in some water-soluble chemical composition, resulting in the increasing rates of fiber on DM basis. Additional studies are necessary to consider the loss ratios of various chemical components in stover during field exposure.

Corn is a high-energy-value crop with good ensiling characteristics due to its relatively high DM content at harvest, low buffering capacity, and adequate WSC content [4,5]. Forage corn silage has the potential to support higher animal performance in ruminant production systems than more conventional conserved forages used in many countries [7]. Fresh corn stover has similar ensiling characteristics as forage corn and can be used to prepare high-quality silage. Corn stover silage has become the major forage component in the diets of dairy cows under most dietary regimes [2,6]. In this study, the fresh corn stover silages of three cultivars were preserved well and of good quality. Conversely, silage prepared from stover that had been exposed for 180 d exhibited poor fermentation quality. The factors involved in assessing fermentation include the chemical composition of the stover materials and the microbial population of epiphytic microorganisms. Fresh stover has a relatively high WSC content and LAB count, and LAB fermented enough sugar to produce lactic acid (Tables 3, 4). In addition, the pH of the silage dropped below 4.0, which inhibited butyric fermentation and ammonia-N production by clostridia. Conversely, silage prepared with stover that had been exposed for 180 d had relatively high pH values and low lactic acid contents. Reductions in sugar and LAB were factors contributing to poor fermentation, as evidenced by the fact that the fresh stover contained abundant sugars as a substrate for LAB to produce lactic acid. Furthermore, as dominant microbes, natural LAB could ferment silage well. Therefore, fresh stover with sufficient sugar content and epiphytic LAB counts, even without LAB inoculation, can be used to make good-quality silage. In a previous study, the natural strains *Lactobacillus casei* and *Lactobacillus plantarum* were the species most frequently isolated from corn stover [24] and can produce relatively high amounts of lactic acid under high WSC conditions compared to other strains.

The CP contents of fresh stover and the resulting silage did not differ greatly, indicating that no protein loss occurred during ensiling, and showed that fresh stover silage could effectively preserve the chemical composition of stover during fermentation. In this study, the natural LAB and ensiling characteristics were compatible with corn stover fermentation and were suitable for producing silage. This indicates that when sufficient natural LAB are present on stover materials, it is unnecessary to employ LAB inoculation to produce silage, and future experiments should study the relationships between stover, LAB characteristics, and silage fermentation. Overall, preparing silage from fresh stover has the benefit of promoting the propagation of natural LAB and inhibiting clostridia growth, as well as inhibiting CP loss during fermentation. From the perspective of microbial populations, chemical composition, and silage fermentation, corn stover silage should be prepared immediately after harvesting corn. Ensilage provides an effective means of conserving summer-grown green forage to supply as winter feed for ruminants [25].

Corn stover can provide some of the metabolizable energy and CP required by ruminants but has the disadvantage of poor intake by ruminants when supplied in a hard, dry state due to the low moisture content and large particle size, making it difficult for ruminants to chew. In addition, low intake and subsequent weight gain have indicated that dry stover is not a preferred forage [1]. In this study, the best and worst results for GP, total VFA content, and OM-D in the stover and silages of the three cultivars were observed for the initial (0 d) and final (180 d) exposure times, respectively. This may have been due to a combined effect of LAB and WSC in fresh stover in improving the fermentation quality, reducing protein loss, and providing more digestible substrates for fermentation by rumen microbes, which could facilitate ruminal digestion. Meanwhile, the microbial population varied among the stover and silage from the three cultivars. Epiphytic microbes include both harmful and beneficial microbes. Consuming such microbes with silage could change the microbial diversity and activity in rumen, affecting rumen digestion. Therefore, good fermentation can inhibit molds growth in silage, and improve the nutrient digestibility of fresh corn stover. Generally, corn stover is beneficial to cattle or sheep producers because it can provide a cheap, locally available feed source [1]. Therefore, further experiments are needed to elucidate alternative feeding strategies for cattle and sheep producers by comparing the nutritional values of diets based on corn stover silage with conventional hay-based diets and assessing the effect on local livestock production systems.

The results confirmed that the silage prepared from fresh corn stover can have beneficial synergistic effects by improving the fermentation quality and promoting ruminal degradation compared to dry corn stover.

## CONCLUSION

Fresh corn stover contained a relatively high LAB count and WSC content, and the resulting silage fermented well, with minimal nutrient loss and improved *in vitro* digestibility. With increasing field exposure of corn stover, the CP and WSC contents and *in vitro* digestibility decreased. From the perspective of microbial population, chemical composition, and silage fermentation, fresh corn stover has suitable ensiling characteristics, and silage should be prepared immediately after harvesting corn.

## Figures and Tables

**Table 1:** Chemical composition of corn stover during field exposure

Exposure (d)	DM (%)	OM	CP	EE	NDF	ADF	WSC

		------------------------------------------------------------% of DM------------------------------------------------------------					
Jinnuo							
0	30.21^f^	93.36^c^	7.99^a^	2.34^a^	59.06^a^	34.70^b^	13.04^a^
7	36.42^e^	94.31^b^	7.64^b^	2.23^a^	53.29^c^	27.44^g^	12.84^b^
15	47.46^b^	93.99^b^	7.43^bc^	2.11^a^	45.46^f^	27.74^f^	12.21^c^
30	55.73^a^	96.02^a^	7.22^cd^	1.98^a^	43.54^g^	29.17^e^	12.32^c^
60	42.41^d^	94.20^b^	7.09^ed^	1.94^a^	58.02^b^	33.53^c^	10.87^d^
90	47.73^b^	93.91^b^	6.85^e^	1.90^a^	49.72^e^	33.05^d^	9.57^e^
180	44.61^c^	93.87^b^	4.41^f^	1.87^a^	50.42^d^	35.21^a^	7.13^f^
Jintian							
0	30.67^g^	94.46^d^	9.51^a^	2.16^a^	45.59^f^	30.50^c^	13.21^a^
7	34.46^f^	95.31^b^	8.28^b^	2.04^ab^	46.00^ef^	32.08^b^	13.05^b^
15	35.25^e^	96.77^a^	8.18^b^	1.90^ab^	46.32^e^	29.09^d^	12.78^c^
30	46.73^a^	95.14^bc^	7.94^c^	1.79^ab^	51.66^c^	26.26^e^	12.24^d^
60	39.42^d^	94.91^c^	7.13^d^	1.75^ab^	49.63^d^	34.64^a^	10.44^e^
90	45.54^b^	95.37^b^	6.33^e^	1.65^ab^	52.50^b^	29.19^d^	9.43^f^
180	43.68^c^	94.58^d^	3.28^f^	1.60^b^	54.65^a^	34.74^a^	7.21^g^
Xianyu							
0	30.43^e^	94.68^e^	6.74^a^	1.55^a^	60.69^a^	34.80^c^	11.75^a^
7	44.78^d^	95.83^b^	6.28^b^	1.46^ab^	51.99^f^	31.55^e^	11.18^b^
15	44.62^e^	95.19^d^	5.58^c^	1.30^ab^	54.57^d^	29.86^f^	10.52^c^
30	54.45^a^	96.21^a^	5.40^c^	1.23abc	56.15^b^	35.88^b^	10.10^d^
60	48.52^c^	94.71^e^	4.59^d^	1.18abc	54.86^cd^	36.15^b^	8.82^e^
90	49.74^b^	95.49^c^	4.43^d^	1.12^bc^	53.63^e^	32.97^d^	7.47^f^
180	48.42^c^	95.38^cd^	3.63^e^	0.85^c^	55.21^c^	37.83^a^	6.30^g^
SEM	0.12	0.08	0.16	0.16	0.11	0.11	0.04
Cultivar (C) means							
Jinnuo	43.51^b^	94.24^c^	6.95^b^	2.05^a^	51.36^b^	31.55^b^	11.14^b^
Jintian	39.39^c^	95.22^a^	7.24^a^	1.84^b^	49.48^c^	30.93^c^	11.19^a^
Xianyu	45.85^a^	94.95^b^	5.24^c^	1.24^c^	55.30^a^	34.15^a^	9.45^c^
Exposure (d, D) means							
0	30.44^g^	93.21^e^	8.08^a^	2.02^a^	55.11^a^	33.33^c^	12.67^a^
7	38.55^f^	95.15^b^	7.40^b^	1.91^ab^	50.43^e^	30.36^e^	12.36^b^
15	42.44^e^	95.32^b^	7.06^c^	1.77abc	48.78^f^	28.9^f^	11.84^c^
30	52.30^a^	95.79^a^	6.85^d^	1.67bcd	50.45^e^	30.44^e^	11.55^d^
60	43.45^d^	94.61^d^	6.27^e^	1.62^cd^	54.17^b^	34.77^b^	10.04^e^
90	47.67^b^	94.93^c^	5.87^f^	1.56^cd^	51.95^d^	31.74^d^	8.82^f^
180	45.57^c^	94.61^d^	3.77^g^	1.44^d^	53.43^c^	35.93^a^	6.88^g^
Significance of main effects and interaction							
C	<0.0001	<0.0001	<0.0001	<0.0001	<0.0001	<0.0001	<0.0001
D	<0.0001	<0.0001	<0.0001	0.0006	<0.0001	<0.0001	<0.0001
C×D	<0.0001	<0.0001	<0.0001	0.9999	<0.0001	<0.0001	<0.0001

DM, dry matter; OM, organic matter; CP, crude protein; EE, ether extract; NDF, neutral detergent fiber; ADF, acid detergent fiber; WSC, water-soluble carbohydrate; SEM, standard error of the mean.

a–g Means within a column with different superscripts differ (p<0.05).

**Table 2:** Microbial population of corn stover during field exposure

Exposure (d)	LAB	Aerobic bacteria	Yeasts	Molds

	---------------------- log_10_ cfu/g of FM -----------------			
Jinnuo				
0	5.25^a^	6.81^cb^	5.76^c^	5.83^a^
7	5.15^ab^	7.03^b^	5.63^cd^	5.42^bc^
15	5.03^b^	7.56^a^	5.45^d^	5.35^c^
30	4.85^c^	6.73^c^	5.46^d^	5.21^c^
60	4.76^cd^	6.45^d^	5.23^e^	5.78^a^
90	4.31^e^	5.31^e^	6.23^b^	5.61^ab^
180	4.62^d^	4.56^f^	6.72^a^	4.57^d^
Jintian				
0	5.13^b^	7.21^b^	5.41^b^	5.62^a^
7	4.82^c^	6.83^c^	5.67^a^	5.26^b^
15	4.53^d^	7.57^a^	3.58^e^	5.12^c^
30	4.42^d^	6.58^d^	4.52^c^	5.05^c^
60	5.24^b^	5.73^e^	5.72^a^	5.62^a^
90	4.52^d^	5.72^e^	4.13^d^	5.37^b^
180	5.41^a^	2.65^f^	3.52^e^	4.57^d^
Xianyu				
0	4.52^b^	8.84^a^	4.67^c^	5.59^a^
7	4.31^c^	6.43^d^	4.31^d^	5.31^b^
15	5.21^a^	6.73^c^	5.23^b^	5.28^b^
30	5.16^a^	7.47^b^	3.52^e^	5.27^b^
60	3.65^d^	6.35^d^	5.32^b^	5.57^a^
90	3.37^e^	5.42^e^	5.93^a^	5.28^b^
180	3.42^e^	6.42^d^	5.73^a^	5.14^b^
SEM	0.05	0.07	0.07	0.06
Cultivar (C) means				
Jinnuo	4.85^a^	6.35^b^	5.78^a^	5.40^a^
Jintian	4.87^a^	6.04^c^	4.65^c^	5.23^b^
Xianyu	4.23^b^	6.81^a^	4.96^b^	5.35^a^
Exposure (d, D) means				
0	4.97^a^	7.62^a^	5.28^bc^	5.68^a^
7	4.76^b^	6.76^d^	5.20^c^	5.33^bc^
15	4.92^a^	7.29^b^	4.75^d^	5.25^cd^
30	4.81^b^	6.93^c^	4.50^e^	5.18^d^
60	4.55^c^	6.18^e^	5.42^a^	5.66^a^
90	4.07^d^	5.48^f^	5.43^a^	5.42^b^
180	4.48^c^	4.54^g^	5.32^ab^	4.76^e^
Significance of main effects and interaction				
C	<0.0001	<0.0001	<0.0001	<0.0001
D	<0.0001	<0.0001	<0.0001	<0.0001
C×D	<0.0001	<0.0001	<0.0001	<0.0001

The samples stored for 60 d and 180 d was soaked by rain before sampling.

LAB, lactic acid bacteria; cfu, colony-forming unit; FM, fresh matter; SEM, standard error of the mean.

a–g Means within a column with different superscripts differ (p<0.05).

**Table 3:** Microbial population of corn stover silages at 60 d of ensiling

Exposure (d)	LAB	Aerobic bacteria	Yeasts	Molds

	----------------------log10 cfu/g of FM-----------------			
Jinnuo				
0	7.26^b^	<10^3^	4.81^c^	ND
7	7.86^a^	<10^3^	4.53^d^	ND
15	7.21^b^	<10^3^	3.13^e^	ND
30	7.02^c^	3.74^b^	4.82^c^	ND
60	6.73^d^	5.64^a^	4.38^d^	ND
90	6.85^d^	<10^3^	7.10^b^	ND
180	6.75^d^	<10^3^	7.83^a^	ND
Jintian				
0	7.16^b^	7.68^a^	3.47^d^	ND
7	7.42^a^	6.47^b^	4.21^b^	ND
15	6.52^d^	6.35^b^	<10^3^	ND
30	6.41^ed^	<10^3^	3.82^c^	ND
60	6.31^e^	3.16^d^	4.46^a^	ND
90	6.50^d^	4.73^c^	4.23^b^	ND
180	6.73^c^	<10^3^	<10^3^	ND
Xianyu				
0	6.52^b^	7.68^a^	3.82^e^	ND
7	7.46^a^	3.15^f^	6.20^b^	ND
15	7.53^a^	5.36^b^	4.56^d^	ND
30	6.02^c^	<10^3^	<10^3^	ND
60	5.28^d^	3.36^e^	6.78^a^	ND
90	4.46^e^	3.78^d^	5.41^c^	ND
180	4.32^e^	4.65^c^	5.38^c^	ND
SEM	0.09	0.09	0.05	-
Cultivar (C) means				
Jinnuo	7.10^a^	<10^3^	5.23^a^	-
Jintian	6.72^b^	4.63^a^	3.58^c^	-
Xianyu	5.94^c^	4.41^b^	4.88^b^	-
Exposure (d, D) means				
0	6.98^b^	5.79^a^	4.03^d^	-
7	7.58^a^	3.59^d^	4.98^c^	-
15	7.09^b^	4.36^b^	3.39^f^	-
30	6.48^c^	3.09^f^	3.55^e^	-
60	6.11^d^	4.05^c^	5.21^b^	-
90	5.94^e^	3.23^e^	5.58^a^	-
180	5.93^e^	<10^3^	5.19^b^	-
Significance of main effects and interaction				
C	<0.0001	<0.0001	<0.0001	-
D	<0.0001	<0.0001	<0.0001	-
C×D	<0.0001	<0.0001	<0.0001	-

LAB, lactic acid bacteria; cfu, colony-forming unit; FM, fresh matter; ND, not detection; SEM, standard error of the mean.

a–e Means within a column with different superscripts differ (p<0.05).

**Table 4:** Chemical composition of corn stover silages at 60 d of ensiling

Exposure (d)	DM	OM	CP	EE	NDF	ADF	WSC

		----------------------------------------------------------------- % DM --------------------------------------------------					
Jinnuo							
0	33.43^a^	94.11^bc^	7.89^a^	2.36^a^	54.62^b^	31.63^b^	5.87^a^
7	33.89^a^	93.97^c^	7.66^b^	2.21^a^	50.31^c^	24.75^g^	5.21^b^
15	33.32^a^	93.92^c^	7.61^b^	2.13^a^	43.57^f^	25.21^f^	5.12^b^
30	33.48^a^	94.46^b^	7.55^b^	2.01^a^	40.62^g^	26.83^e^	5.15^b^
60	33.74^a^	95.23^a^	7.02^c^	1.96^a^	55.48^a^	30.42^c^	4.65^c^
90	33.27^a^	94.53^b^	7.04^c^	1.93^a^	46.72^e^	30.02^d^	4.13^d^
180	33.89^a^	94.28^bc^	4.35^d^	1.91^a^	47.41^d^	32.57^a^	4.06^d^
Jintian							
0	33.75^a^	94.39^c^	8.58^a^	2.15^a^	40.32^g^	27.63^d^	6.43^a^
7	33.31^a^	94.28^c^	8.13^b^	2.06^ab^	43.64^f^	30.02^c^	6.21^b^
15	33.42^a^	95.57^a^	7.83^c^	1.92^ab^	45.13^e^	26.35^e^	6.14^b^
30	33.35^a^	95.74^a^	7.66^c^	1.81^b^	48.47^c^	23.41^f^	5.79^c^
60	33.74^a^	94.83^b^	7.46^d^	1.76^ab^	46.78^d^	31.68^b^	5.65^d^
90	33.21^a^	94.91^b^	6.72^e^	1.68^ab^	50.24^b^	27.48^d^	5.07^e^
180	33.85^a^	94.57^bc^	3.74^f^	1.59^b^	51.69^a^	32.31^a^	4.43^f^
Xianyu							
0	33.00^a^	93.25^e^	6.10^a^	1.56^a^	54.68^a^	32.52^c^	5.15^a^
7	33.65^a^	93.53^ed^	5.88^a^	1.42^a^	47.52^f^	29.46^d^	5.07^a^
15	33.53^a^	94.36^c^	5.61^b^	1.28^ab^	50.68^e^	26.73^e^	4.32^b^
30	33.63^a^	95.28^b^	5.30^c^	1.22^b^	53.76^b^	32.75^c^	4.12^c^
60	33.52^a^	95.95^a^	5.27^c^	1.17^ab^	52.94^c^	33.82^b^	4.11^c^
90	33.21^a^	94.37^c^	5.17^c^	1.15^ab^	51.32^d^	29.73^d^	4.04^c^
180	33.68^a^	93.59^d^	3.58^d^	0.89^b^	54.51^a^	35.25^a^	4.02^c^
SEM	0.35	0.07	0.14	0.16	0.1	0.12	0.04
Cultivar(C) means							
Jinnuo	33.57^a^	94.36^b^	7.02^b^	2.07^a^	48.39^b^	28.78^b^	4.88^b^
Jintian	33.52^a^	94.90^a^	7.16^a^	1.85^b^	46.61^c^	28.41^c^	5.67^a^
Xianyu	33.46^a^	94.33^b^	5.27^c^	1.24^c^	52.20^a^	31.47^a^	4.40^c^
Exposure (d, D) means							
0	33.39^a^	93.92^d^	7.52^a^	2.02^a^	49.87^c^	30.59^c^	5.82^a^
7	33.62^a^	93.93^d^	7.22^b^	1.90^ab^	47.16^f^	28.08^e^	5.50^b^
15	33.42^a^	94.62^b^	7.02^c^	1.78abc	46.46^g^	26.10^g^	5.19^c^
30	33.49^a^	95.16^a^	6.84^d^	1.68bcd	47.62^e^	27.66^f^	5.02^d^
60	33.67^a^	95.342^a^	6.58^e^	1.63bcd	51.73^a^	31.97^b^	4.80^e^
90	33.23^a^	94.60^b^	6.31^f^	1.59^cd^	49.43^d^	29.08^d^	4.41^f^
180	33.81^a^	94.15^c^	3.89^g^	1.46^d^	51.20^b^	33.38^a^	4.17^g^
Significance of main effects and interaction							
C	0.8274	<0.0001	<0.0001	<0.0001	<0.0001	<0.0001	<0.0001
D	0.483	<0.0001	<0.0001	0.0017	<0.0001	<0.0001	<0.0001
C×D	0.9683	<0.0001	<0.0001	0.9999	<0.0001	<0.0001	<0.0001

DM, dry matter; OM, organic matter; CP, crude protein; EE, ether extract; NDF, neutral detergent fiber; ADF, acid detergent fiber; WSC, water-soluble carbohydrate; SEM, standard error of the mean.

a–g Means within a column with different superscripts differ (p<0.05).

**Table 5:** Fermentation quality of corn stover silages at 60 d of ensiling

Exposure (d)	pH	Lactic acid (% of FM)	Acetic acid (% of FM)	Propionic acid (% of FM)	Butyric acid (% of FM)	Ammonia-N (g/kg of FM)
Jinnuo						
0	3.76^d^	1.83^a^	0.58^c^	0.08^c^	ND	0.60^f^
7	3.90^d^	1.07^bc^	0.30^d^	0.07^c^	ND	0.55^f^
15	4.29^c^	0.89^c^	0.97^b^	0.07^c^	0.02^a^	1.42^e^
30	4.38^bc^	1.79^a^	0.98^b^	0.11^c^	0.02^a^	3.56^a^
60	4.47^b^	1.11^bc^	1.50^a^	0.34^b^	0.01^ab^	2.95^c^
90	4.35^bc^	1.23^b^	0.81^b^	0.15^c^	ND	2.52^d^
180	6.63^a^	0.94^c^	0.42^cd^	0.46^a^	0.01^ab^	3.37^b^
Jintian						
0	3.84^d^	2.53^a^	0.42^c^	0.09^d^	0.01^ab^	0.37^d^
7	4.07^c^	2.43^a^	0.86^b^	0.11^d^	0.02^a^	0.42^d^
15	4.25^b^	0.83^d^	0.42^c^	0.37^b^	0.02^a^	0.67^c^
30	4.35^b^	0.58^e^	0.53^c^	0.20^c^	ND	1.41^b^
60	4.28^b^	1.32^c^	1.17^a^	0.53^a^	0.01^ab^	1.30^b^
90	4.32^b^	1.52^b^	1.20^a^	0.09^d^	0.01^ab^	2.33^a^
180	5.64^a^	0.85^d^	0.41^c^	0.51^a^	ND	2.40^a^
Xianyu						
0	4.07^c^	1.06^b^	0.68^bc^	ND	ND	0.67^e^
7	4.04^c^	1.36^a^	0.64^bc^	ND	ND	0.62^e^
15	4.28^b^	0.89^cd^	0.55^c^	0.07^cd^	0.01^ab^	1.52^d^
30	4.43^b^	0.79^de^	0.74^ab^	0.13^bc^	ND	2.43^c^
60	4.36^b^	0.64^f^	0.87^a^	0.30^a^	0.01^ab^	2.65^b^
90	4.39^b^	0.95^c^	0.56^c^	0.06^cd^	ND	2.70^b^
180	6.31^a^	0.73^ef^	0.61^bc^	0.20^b^	0.02^a^	3.25^a^
SEM	0.05	0.05	0.05	0.03	0.0049	0.06
Cultivar (C) means						
Jinnuo	4.54^a^	1.27^b^	0.79^a^	0.18^b^	0.01^a^	2.14^a^
Jintian	4.39^b^	1.44^a^	0.72^b^	0.27^a^	0.01^a^	1.27^c^
Xianyu	4.55^a^	0.92^c^	0.66^b^	0.11^c^	0.01^a^	1.98^a^
Exposure (d, D) means						
0	3.89^e^	1.81^a^	0.56^ed^	0.06^d^	0.00^b^	0.55^e^
7	4.00^d^	1.62^b^	0.6^d^	0.06^d^	0.01^b^	0.53^e^
15	4.27^c^	0.87^e^	0.65^d^	0.17^b^	0.02^a^	1.20^d^
30	4.39^b^	1.05^d^	0.75^c^	0.15^bc^	0.01^b^	2.47^b^
60	4.37^b^	1.02^d^	1.18^a^	0.39^a^	0.01^ab^	2.30^c^
90	4.35^bc^	1.23^c^	0.86^b^	0.10^cd^	0.00^b^	2.52^b^
180	6.19^a^	0.84^e^	0.48^e^	0.39^a^	0.01^ab^	3.01^a^
Significance of main effects and interaction						
C	<0.0001	<0.0001	0.0002	<0.0001	0.2579	<0.0001
D	<0.0001	<0.0001	<0.0001	<0.0001	0.0247	<0.0001
C×D	<0.0001	<0.0001	<0.0001	<0.0001	0.0038	<0.0001

FM, fresh matter; ND, not detection; SEM, standard error of the mean.

a–e Means within a column with different superscripts differ (p<0.05).

**Table 6:** Ruminal pH, gas production, total VFA content and OM-D of corn stover and silages at 60 d of ensiling

Exposure (d)	Corn stover				Corn stover silage			
	
	Ruminal pH	GP (mL/g DM)	Total VFA (mmol/L)	OM-D (%)	Ruminal pH	GP (mL/g DM)	Total VFA (mmol/L)	OM-D (%)
Jinnuo								
0	6.15^c^	44.12^a^	40.47^a^	40.10^a^	6.53^d^	49.32^a^	51.21^a^	46.42^a^
7	6.18^bc^	43.95^a^	40.05^ab^	39.84^a^	6.61^cd^	49.03^a^	50.14^ab^	45.34^a^
15	6.19^bc^	44.02^a^	39.58^ab^	38.43^b^	6.65^bc^	47.22^b^	49.24^bc^	44.76^ab^
30	6.23abc	42.56^a^	38.42^b^	38.15^b^	6.70^bc^	46.52^bc^	49.05^bc^	43.25^bc^
60	6.25^ab^	40.23^b^	36.14^c^	36.43^c^	6.73^b^	46.02bcd	48.46^c^	43.06^c^
90	6.28^a^	39.21^b^	35.46^c^	35.21^d^	6.84^a^	45.21^cd^	44.47^d^	43.02^c^
180	6.30^a^	35.46^c^	32.10^d^	33.16^e^	6.88^a^	44.28^d^	41.10^e^	41.16^d^
Jintian								
0	6.12^b^	45.21^a^	41.25^a^	40.19^a^	6.64^e^	50.04^a^	53.86^a^	47.25^a^
7	6.18^ab^	45.16^a^	40.56^ab^	39.86^a^	6.67^de^	49.61^a^	52.61^ab^	47.14^a^
15	6.21^ab^	44.68^a^	40.21^ab^	38.43^b^	6.71cde	48.72^ab^	52.46^b^	47.06^a^
30	6.23^a^	43.27^b^	39.72^b^	36.72^c^	6.74bcd	47.81^b^	50.72^c^	46.84^a^
60	6.26^a^	42.81^b^	37.62^c^	34.16^d^	6.80^bc^	46.26^c^	48.25^d^	45.23^b^
90	6.25^a^	41.53^c^	36.41^d^	33.71^d^	6.83^b^	45.87^c^	45.65^e^	43.64^c^
180	6.27^a^	38.24^d^	34.71^e^	31.72^e^	6.93^a^	44.18^d^	42.19^f^	41.30^d^
Xianyu								
0	6.19^a^	43.51^a^	38.54^a^	39.82^a^	6.50^e^	47.26^a^	50.12^a^	46.29^a^
7	6.20^a^	43.16^ab^	38.69^a^	38.43^ab^	6.56^de^	47.03^a^	49.26^ab^	46.11^a^
15	6.21^a^	42.28^b^	36.27^b^	37.13^bc^	6.64^cd^	46.14^ab^	49.01^ab^	46.08^a^
30	6.22^a^	39.84^c^	35.43^b^	35.41^cd^	6.71^bc^	45.67abc	47.83^b^	44.26^b^
60	6.24^a^	39.27^cd^	33.76^c^	34.35^de^	6.76^ab^	45.24^bc^	45.76^c^	41.34^c^
90	6.25^a^	38.46^d^	31.84^d^	32.85^e^	6.81^ab^	44.29^c^	42.08^d^	40.62^c^
180	6.26^a^	36.73^e^	30.73^d^	30.73^f^	6.86^a^	42.16^d^	38.65^e^	38.86^d^
SEM	0.03	0.40	0.46	0.48	0.03	0.53	0.46	0.47
Cultivar (C) means								
Jinnuo	6.23^a^	41.36^b^	37.46^b^	37.33^a^	6.71^b^	46.80^b^	47.67^b^	43.86^b^
Jintian	6.22^a^	42.99^a^	38.64^a^	36.40^b^	6.76^a^	47.50^a^	49.39^a^	45.49^a^
Xianyu	6.22^a^	40.46^c^	35.04^c^	35.53^c^	6.69^b^	45.40^c^	46.10^c^	43.37^b^
Exposure (d, D) means								
0	6.15^d^	44.28^a^	40.09^a^	40.04^a^	6.56^f^	48.87^a^	51.73^a^	46.65^a^
7	6.19^cd^	44.09^a^	39.77^a^	39.38^a^	6.61^e^	48.56^a^	50.67^b^	46.20^a^
15	6.20bcd	43.66^a^	38.69^b^	38.00^b^	6.67^d^	47.36^b^	50.24^b^	45.97^a^
30	6.23abc	41.89^b^	37.86^c^	36.76^c^	6.72^cd^	46.67^bc^	49.2^c^	44.78^b^
60	6.25^ab^	40.77^c^	35.84^d^	34.98^d^	6.76^c^	45.84^cd^	47.49^d^	43.21^c^
90	6.26^a^	39.73^d^	34.57^e^	33.92^e^	6.83^b^	45.12^d^	44.07^e^	42.43^d^
180	6.27^a^	36.81^e^	32.51^f^	31.87^f^	6.89^a^	43.54^e^	40.65^f^	40.44^e^
Significance of main effects and interaction								
C	0.8004	<0.0001	<0.0001	<0.0001	0.0004	<0.0001	<0.0001	<0.0001
D	<0.0001	<0.0001	<0.0001	<0.0001	<0.0001	<0.0001	<0.0001	<0.0001
C×D	0.927	0.0028	0.0816	0.1884	0.7522	0.7809	0.0647	0.0004

VFA, volatile fatty acid; OM-D, *in vitro* OM degradability for 48h incubation; GP, gas production for 48h incubation; SEM, standard error of the mean.

a–f Means within a column with different superscripts differ (p<0.05).
